# Which Temporal Difference learning algorithm best reproduces dopamine activity in a multi-choice task?

**DOI:** 10.1186/1471-2202-14-S1-P144

**Published:** 2013-07-08

**Authors:** Jean Bellot, Mehdi Khamassi, Olivier Sigaud, Benoît Girard

**Affiliations:** 1 Université Pierre et Marie Curie, Institut des Systemes Intelligents et de Robotique, Paris, France; 2 Centre National de la Recherche Scientifique, UMR 7222, Paris, France

## 

The activity of dopaminergic (DA) neurons has been hypothesized to encode a reward prediction error (RPE) [1] which corresponds to the error signal in Temporal Difference (TD) learning algorithms [2]. This hypothesis has been reinforced by numerous studies showing the relevance of TD learning algorithms to describe the role of basal ganglia in classical conditioning. However, most studies have recorded DA activity during pavlovian conditioning, and thus the exact nature of the signal encoded by DA neurons during a choice remains unclear. In the literature of reinforcement learning different TD learning algorithms predict different RPE during a choice. If the algorithm SARSA predicts a RPE based on the future choice, Q-learning predicts a RPE that will be based on the action that maximize the future amount of reward and V-learning predicts a RPE based on an average of the values of the different available options.

Recent recordings of DA neurons during multi-choice tasks investigated this issue and raised contradictory interpretations on whether DA's RPE signal is action dependent [3] or not [4]. While the first study suggests that DA neurons encode a RPE compatible with SARSA, results from the second study are interpreted as more consistent with Q-learning [4]. However these studies only proposed a qualitative comparison of the ability of these TD learning algorithms to explain these patterns of activity. In this work we simulated and precisely analyzed these algorithms in relation with previous electrophysiological recordings in a multi-choice task performed by rats [4]. We found that, when fitting the behavior, the simulated algorithms predict a fast convergence of the RPE, incompatible with the observed DA activity, suggesting an apparent dissociation between the signal encoded by dopamine neurons and behavioral adaption of the animals. Further analyses of the evolution of dopamine neurons activity across learning indicated that, complementarily to the RPE, the value function fits well with the activity. However the value function cannot explain the inhibition of DA activity during omission and the global decrease of DA activity during a session at the time of reward delivery. Thus in this task, information about both RPE and value may be conveyed by dopamine activity.

By quantitatively comparing the ability of the different TD learning algorithms, this work shows the limitation of these algorithms to fit both the behavior and the DA activity observed in a multi-choice task, when interpreting DA activity as a RPE only. Unexpectedly we show that a value function better fits DA activity suggesting that DA neurons recorded in this task may encode multiple information.
